# Affording reusable data: recommendations for researchers from a data-intensive project

**DOI:** 10.1038/s41597-025-04565-0

**Published:** 2025-02-12

**Authors:** Gorka Fraga-González, Hester van de Wiel, Francesco Garassino, Willy Kuo, Diane de Zélicourt, Vartan Kurtcuoglu, Leonhard Held, Eva Furrer

**Affiliations:** 1 Epidemiology, Biostatistics and Prevention Institute (EBPI) and Center for Reproducible Science (CRS), University of Zurich, Hirschengraben 84, 8001 Zurich, Switzerland; 2 The Interface Group, Institute of Physiology, University of Zurich, Winterthurerstrasse 190, 8057 Zurich, Switzerland

**Keywords:** Research data, Scientific community

## Abstract

Scientists are increasingly required by funding agencies, publishers and their institutions to produce and publish data that are Findable, Accessible, Interoperable and Reusable (FAIR). This requires curatorial activities, which are expensive in terms of both time and effort. Based on our experience of supporting a multidisciplinary research team, we provide recommendations to direct the efforts of researchers towards affordable ways to achieve a reasonable degree of “FAIRness” for their data to become reusable upon its publication. The recommendations are accompanied by concrete insights on the challenges faced when trying to implement them in an actual data-intensive reference project.

## Background

Growing efforts to improve scientific reproducibility have produced demands that researchers make their data and code with the corresponding metadata available in dedicated repositories. But unless the metadata are properly handled to enable reusability, the published data will amount to mere “data dumps” of limited utility. While steps towards FAIR data are increasingly mandated by funding bodies, activities related to enabling them are often not sufficiently funded. Yet, producing data that are FAIR to some degree requires intensive curatorial activity, that is, actions that involve organizing, documenting, and integrating data at the different stages of the data life cycle so that it becomes a useful resource. In addition to financing limitations, the costs associated with these curation tasks can be difficult for researchers to estimate a priori. Factors such as ambiguous definitions of the specific tasks involved, institutional complexity or variability of funding sources may affect both estimations and effective costs^1^, challenging the establishment of realistic budgets for research proposals.

The AFFORD project (*A Framework for Avoiding the Open Research Data Dump*) at the University of Zurich (UZH) followed a reference project in the area of biomedicine to study the challenges associated with data curation in an interdisciplinary, multi-institutional and data-intensive environment. The experience we gained from supporting the researchers of this reference project allowed us to formulate a support framework that could be extended to other researchers and institutions. In this report, we provide recommendations for researchers and insights based on our experience in AFFORD and its reference project.

### A FAIRly common goal

The FAIR principles - Findability, Accessibility, Interoperability and Reusability^2^ - constitute the most widely adopted framework for guiding the management of digital resources, in particular, scientific data and metadata. Although open to interpretation and not exempt from ambiguity, they define guiding principles and practices intended to enable both machines and humans to reuse research data. These core precepts have led to a myriad of guidelines and initiatives to define, evaluate and implement FAIR principles in practice. The challenges and considerations associated with the implementation of each principle are discussed in detail in a previous report^3^. To address the diversity of practical implementations, the “FAIR data maturity model” working group proposed a set of criteria to monitor FAIRness maturity levels^4^, further highlighting that there is no universal approach for implementing FAIR data in life sciences, nor a broad consensus within the community on a concrete implementation for any specific data type. The support activities and recommendations in this report are heavily inspired by some recent technical guidelines for the *FAIRification* of research data^3,5^. The flexible *FAIRification* framework^5^, in particular, emphasizes three key aspects for practical implementation: setting realistic, incremental goals; tailoring support to the individual needs; and working in multidisciplinary teams, involving all scientific and infrastructure stakeholders relevant to the individual project. The authors suggest aiming for a balanced “FAIR enough” status of the data, depending on the available resources and required capabilities^5^.

In effect, the goal of the FAIR principles is not to enforce adherence to a set of rigid rules, but rather to promote the generation of reusable scientific data and metadata, thereby advancing knowledge. Nonethless, some researchers may be hesitant to engage in “FAIRification” activities if they perceive that adhering to the FAIR principles might detract from producing outputs with more immediate scientific rewards, such as journal publications. Fortunately (or rather hopefully), the culture of considering research articles as a primary research output is evolving, driven in part by the increasing pressure from institutions, journals and funding agencies to ensure data availability in repositories and to include data management plans in funding applications. Several initiatives are promoting this cultural change. For example, the San Francisco Declaration on Research Assessment (Dora; https://sfdora.org), initiated in 2012, calls for a stop to the use of journal impact factors and other quantitative indicators in evaluating research. Recently, the DORA consortium launched an online tool with examples of alternative research assessment implementations. Along similar lines, the Coalition for Advancing Research Assessment (CoARA; https://coara.eu) aims to reform research assessment, promoting quality over quantity. To enable this transition, the “Responsible Research Assessment” initiative^6^ proposed an actionable evaluation framework called RESQUE (RESearch QUality Evaluation). Collectively, these declarations and initiatives, endorsed by multiple universities and scientific associations, reflect a growing commitment to reforming research assessment practices with a stronger focus on quality and FAIRness.

In addition to considerations related to research assessment and publications, managing data according to FAIR principles–as if intended for public access–provides direct benefits, including improved knowledge transfer and reproducibility within the research group. These factors are particularly critical given the frequent turnover of research personnel, especially students and postdoctoral researchers. While the initial investments in time and effort for data management activities may be substantial, these costs decrease over time and are ultimately outweighted by the benefits of having curated, FAIR data. The recommendations and insights provided in this report are intended to help lower the initial costs.

### Concurrent vs post-hoc data management

Data management activities that are *concurrent* to the research output production can be much more efficient and easier to implement than if they are performed *post-hoc*, i.e., after data production. The obvious course of action is to encourage researchers to have data management plans (DMPs), as is, for example, mandated for projects funded by the Swiss National Science Foundation. For these DMPs to be implementable and not turn into empty promises, funding bodies should also cover at least some of the costs incurred in their implementation^7^. Even then, there are frequent scenarios in which researchers will need to apply *post-hoc* data management. For instance, in time-sensitive experiments, only limited time resources may be available and a compromise will have to be found between concurrency and scope of the data curation. In some cases, researchers may need to work with data already produced by someone else, e.g., if they join a project in an ongoing data production phase. Metadata is often incomplete and valuable time is spent finding, reorganizing, or documenting the data. At times, researchers find themselves in situations where the only way to obtain the metadata is to contact researchers formerly involved in data acquisition. However, this is not always possible, and sometimes even those who collected the data do not accurately remember the details of the data acquisition. The recommendations and insights provided in this report should also be helpful in such suboptimal situations.

### The reference project

The reference project followed by AFFORD involved four research groups with different areas of expertise and from three different Swiss universities who joined forces to study elements of the mouse central nervous system using in-vivo, ex-vivo and in silico approaches and experimental facilities on four continents. Among other techniques, the researchers utilized imaging modalities such as synchrotron radiation-based micro computed tomography and multi-photon fluorescence microscopy, producing on the order of 1 petabyte of data. The research output also included software, computational models, protocols and auxiliary hardware. Notably, the setup of the reference project included a specific commitment to generate reusable data and metadata, which was to be fulfilled with the help of AFFORD.

The AFFORD project, funded independently from the reference project, is a partnership between one of the reference project’s research groups (The Interface Group from the Institute of Physiology), and the Center for Reproducible Science (CRS), both at UZH. With the diversity of methods and data types involved, the reference project of AFFORD captures some of the most important practical challenges faced by research groups, irrespective of their scientific field. Similarly, the support experience gained through AFFORD offers a good example of common issues that data-oriented support staff may encounter when interacting with researchers from different disciplines.

### An affordable approach

Although data stewardship infrastructure is increasingly provided by academic institutions, researchers still have to find ways of producing “FAIR-enough” data using their own resources and considering the time frame of their careers. Therefore, our focus is on prioritizing practices, workflows and tools that can be realistically integrated in a laboratory’s routine without creating new dependencies or incurring unsustainable costs. These practices do not necessarily make the data FAIR to a high degree, but they are essential steps towards enabling FAIRification. As a bonus, many of these practices can lead to gains in research efficiency for the individual researchers beyond data curation.

Based on the experience gained through the AFFORD’s reference project, we provide here a set of key actions and general recommendations to facilitate the creation of reusable data, followed by examples and challenges faced during their implementation in the reference project. We dedicate a section to each of the following key actions: Providing metadata (Section 1), Sharing metadata efficiently with internal and external collaborators (Section 2), and Archiving and publishing data (Section 3). The recommendations for each key action are summarized in Table 1 and the most relevant technical resources for each key action are summarized in Table 2.

**Table 1:** Summary of the key actions and recommendations to produce better reusable research data.

Key Actions and recommendations	
1.	Providing metadata
	• Adopt existing standards as much as possible
	• Build upon previous work and collaborate
	• Keep past versions and assess risks of introducing changes
	• Make machine-readable spreadsheets
	• Learn how to perform table operations with code
	• Keep text formatting to a minimum
	• Keep the workflow as simple and consistent as possible and as flexible as necessary
2.	Sharing metadata
	• Choose a secure and version-controlled platform
	• Consider ease of use and practicality of the platform
	• Make a decision at a group or project level
	• Share code efficiently
	• Create off-boarding/ on-boarding documentation
3.	Archiving and publishing
	• Organize data folders to facilitate archiving
	• Start archival curation early on
	• Use specialized utilities for moving or copying files
	• Keep metadata of the storage locations
	• Find a suitable repository
	• Publish with persistent identifiers to ensure long-term findability

**Table 2:** List of the most relevant technical resources recommended in each of the key actions.

Resource	Description
1. Providing metadata	
dplyr	R language library that uses a grammar for data manipulation designed to facilitate data analysis. We recommend it for handling tabular metadata. https://dplyr.tidyverse.org/
fairsharing	Platform to navigate through data and metadata standards from different fields. We recommend it as a starting point for planning metadata and project organization. https://fairsharing.org
JSON	JavaScript Object Notation (JSON). An open standard format for data interchange, which is text-based and consists of name/value pairs and value arrays. We recommend it for the companion metadata files because it is easy to read by humans and by many programming languages. https://www.json.org/json-en.html
Markdown	A lightweight language that uses simple syntax symbols to add formatting elements to plain text. We recommend it for documentation because it is simple and interoperable. https://www.markdownguide.org/
2. Sharing metadata	
Git	A distributed version control system. A Git repository is a set of folders and files with complete history and full version-tracking abilities. We recommend it for maintaining and sharing metadata, code and documentation, but it is not intended for data archiving. https://git-scm.com/
Gitlab	Open-source hosting service for Git repositories. Many research institutions maintain their own instances of Gitlab and provide support for many of its advanced features. https://about.gitlab.com/
Jupyter Notebooks	Open source web application to share interactive documents with code, narrative text and embedded outputs like plots and tables. The source files (.ipynb) are based on JSON format and can be exported in multiple formats. They can be combined with Quarto for more advanced formatting. We recommend them for sharing Python code. https://jupyter.org/
Quarto	Open-source publishing system for multiple languages (e.g., R, Python) and output formats (e.g.,PDF, HTML or MS word). The source files (.qmd) used to generate reports with code, text and rich outputs are written in plain text. We recommend it for dynamic reporting and sharing metadata, code and documentation. https://quarto.org/
3. Archiving and publishing	
re3data	Registry of research data repositories that allows exploring the available options for a variety of domains. We recommend it to find suitable FAIR repositories for data archiving. https://www.re3data.org/

## 1. Providing metadata

The term *metadata* refers to data about data. This includes documents intended for human use, consisting mainly of text and images, as well as machine-readable metadata such as tables and structured metadata files that accompany data. Machine-readable metadata can be easily processed by computers without human intervention. Many researchers create some of these metadata files with the intention of sharing them internally. Often, the metadata is incomplete and lacks information for understanding data, e.g., clear explanations of variable names. Automating some of the metadata generation, for example creating JavaScript Object Notation (JSON) metadata files (see Table 2), can be challenging for some laboratories due to lack of coding expertise. While much of the information can be conveyed in flat, tabular formats (e.g., CSV), metadata files like JSON are needed when dealing with hierarchical or complex information, which is necessary to enable machine actionability of the data when integrating it into more advanced FAIRification systems. Another popular format for such structured metadata files is the Extensible Markup Language (XML), but we refer to JSON in this report as it is considered a more simple and flexible format. The following recommendations are intended to assist researchers in directing their efforts towards generating the essential set of metadata outlined in Table 3.

**Table 3:** Minimum set of metadata as starting point towards reusable data.

Metadata	Description
File naming convention	Documents describing the meaning of data filename parts, folders and their organization.
Procedures	Documents describing acquisition protocols and any preprocessing required to generate the source, reusable data.
READMEs	Plain text file(s) with high-level information about folder contents.
Tables^*^	Tabular metadata describes files, recording parameters, subject or model characteristics, data provenance, data storage location, and any other information relevant to finding, accessing and reusing the data.
Companion files^*^	Files stored adjacent to the data files (e.g., JSON files) with structured metadata about the files they apply to.
Codebooks^*^	Codebooks explain the variable names, units, abbreviations and acronyms used in all metadata files.

^*^Metadata that can be read by both humans and machines.

### Recommendations

The large number of available guidelines, data management tools and metadata standards can overwhelm researchers and discourage curatorial efforts, especially when research is conducted with limited time, data expertise or personnel resources. This, and the lack of continuous support makes it very difficult for many laboratories to adopt advanced workflows and complex tools that enable the production of FAIR data. However, these difficulties should not prevent laboratories from avoiding data dumps and the production of research waste^8^. We suggest a minimum set of metadata that can be provided without much effort, which we consider as the starting point for FAIR data production (see Table 3). This set contains the required files in many online repositories and the basic formatting and content requirements to start FAIRification. When defining this set of minimum metadata, we also considered the capabilities of researchers and the resources frequently available in projects similar to our reference project.

#### Adopt existing standards as much as possible

There are many available metadata standards, examples and templates. We recommend searching for what is available in the respective researcher’s field, reusing it if possible, and modifying it if necessary. A systematic search for field-specific metadata resources can be performed using the fairsharing (https://fairsharing.org/) resource, which has an extensive list of data and metadata standards, databases and policies, and is kept up-to-date by the field-specific research communities^9^. Adopting available standards will result in a much lower workload than defining a new standard from scratch. Therefore, we recommend using actively maintained standards employed by the community of researchers in a domain. This will also ease documentation, refering to conventions the broader community is already familiar with. Field-specific metadata standards usually describe the structure of folders, file naming conventions, and relevant fields in the metadata tables and the companion JSON files. If no standard is available for a specific field, one may look for standards in different fields that use similar data types. If field-specific standards are available but are not directly applicable to a specific project, we recommend following them as much as possible and adapting what is needed to fit the project’s data, preferably after exchanging with colleagues in the field. Some popular and growing standards, such as the Brain Imaging Data Structure (BIDS^10^) offer the possibility of submitting extension proposals for additional data types (or imaging techniques in the case of BIDS^11,12^).

#### Build upon previous work and collaborate

Data curation efforts are collaborative, continuous processes. They should not be undertaken by individuals in isolation, and they can be updated or corrected after multiple re-uses of the data. Like reusing metadata standards, we recommend building on existing internal documentation when available. Importantly, if internal notes need to be created from scratch, expanded, updated or corrected, they should be accessible for future users to build upon them or reuse them. Which modifications would be most useful is an important point to discuss with other potential re-users.

#### Keep past versions and assess the risks of introducing changes in existing metadata

Be aware that other researchers may be using the source data and metadata while changes are being introduced, which may lead to errors. To minimize that risk, all metadata versions should be kept for reference.

#### Make machine-readable spreadsheets

Tabular metadata are used in almost all research fields. Table formats such as comma-separated value (CSV) and tab-separated value (TSV) are usually recommended for the sake of accessibility and interoperability between software applications. However, a much larger fraction of researchers is familiar with using spreadsheet software like Excel or Google spreadsheets than are familiar with proper import/export and encoding of CSV and TSV tables. Fortunately, it is possible to make user-friendly spreadsheets compatible with FAIR data systems^13^. For that, researchers need to produce spreadsheets with some degree of organization and formatting consistency. Guidelines for increasing the machine-readability of spreadsheets are given in^14^ and^15^. From these guidelines, we highlight the need for consistency in formatting and machine-readability to enable automation of data validation, preprocessing, synthesis, and analysis of tabular metadata.

#### Learn how to perform table operations with code

There are a few table operations that are often performed when handling metadata, like joining tables by a key variable, checking table dimensions, looking for entry errors or missing values, reshaping tables, etc. Performing these data checks and manipulations manually is prone to errors, makes modifications hard to trace back and is very impractical for large tables. Note that some errors in metadata handling can be fatal, for example, assigning a subject ID to the wrong recording parameters or assigning the wrong group label to a subject^16^. Most of these operations can be performed with software for spreadsheets like Excel, which has some automation capabilities. However, Excel cannot handle large data sets and makes it cumbersome to integrate table operations into reproducible workflows, version control, as well as advanced visualization and analysis tools. There are some preferable software choices for more accessible, reproducible, and efficient data handling that do not require advanced programming skills. The R package plyr (https://dplyr.tidyverse.org/) offers code for table manipulation that we consider reasonably intuitive and readable for non-advanced programmers. R is free and open source, widely used in research and easy to set up with Integrated Development Environment (IDE) applications like Rstudio (https://posit.co/products/open-source/rstudio/) or VS code (https://code.visualstudio.com/). In short, we strongly recommend a minimum investment into automating table operations. This investment also pays off in activities beyond FAIR data curation, as table operations are often performed for data analysis.

#### Keep text formatting to a minimum

To facilitate accessibility and software interoperability of text documentation, we recommend using plain text files with Markdown (https://www.markdownguide.org/), a lightweight markup language, to specify the structure and formatting of the document. While for small annotations or README files, plain TXT files without formatting can suffice, this can in turn strongly limit the length and type of content that can be shared in a human-readable way. Markdown uses simple syntax symbols (like #, ##, *, **) that will add formatting elements to the text. This syntax is widely used for online technical documentation because Markdown documents are easy to create, they can be read across multiple operating systems without requiring special software, and they can include tables and images (or animations if intended for HTML). Markdown documents can also be rendered into formatted text in multiple file formats that might be required in some instances when sharing documentation and reports among collaborators and institutions (e.g., PDF, DOC, HTML). There is a variety of free and commercial editors that facilitate editing of Markdown documents with user experience similar to word processors like Microsoft Word. These are especially useful when inserting tables and images into documents. In addition to specific software, most IDEs used for coding also have integrated visual Markdown editor functionalities. The constrained formatting options of Markdown also bring the benefit of investing less time in styling documents or fixing formatting errors.

#### Keep the workflow as simple and consistent as possible and as flexible as necessary

If most of the projects in a research group follow consistent approaches for data documentation and metadata filing, the overall overhead associated with the corresponding activities will decrease over time, compliance will increase, and documentation processes will improve. Although consistency is important, flexibility is still necessary to accommodate individual needs and incorporate ad-hoc adaptations to requirements that emerge with new projects. In terms of implementation, all team members should ideally be able to perform all data management tasks. This is especially relevant when considering the relatively short time span of academic research positions: new team members should be able to take over at least the core of the data management activities within a short time. Here, we note that there are advanced lab management tools that enable the management of data and metadata at all stages of the data cycle and their integration into a common space, alongside lab notes, documentation and inventory^17,18^. These tools can be useful solutions for large laboratories or research facilities. However, adapting a research group’s workflow to such a system can be very expensive, particularly in terms of the time required to set up and master the system, in addition to any financial costs associated with using the services. Another concern is the long-term viability and reliance on relatively closed data management systems. Therefore, these tools cannot be considered universally affordable or accessible solutions.

### Implementation in the reference project

As part of the AFFORD project, we aimed to implement the minimum set of metadata outlined in Table 3 in the reference project. Specifically, our initial interactions took place with researchers collecting images with synchrotron radiation-based computed micro tomography at multiple international synchrotron facilities. The researchers collected data sets (each containing many images of a group of mice) in a specific facility and during a specific time period. These synchrotron images are associated with rich and abundant metadata. Our support for metadata curation addressed the following gaps:

- Researchers had no previous knowledge of *metadata standards* specific to their field or data. We proposed BIDS^10^ as a reference, as this covers multiple brain imaging techniques, and is very detailed, broadly used and regularly updated. BIDS was used only as reference, since each synchrotron facility had its own restrictions for naming conventions and folder organization.
- We derived a post-hoc *file naming* scheme for data that was already collected and lacked this documentation. One difficulty was to strike a balance between maintaining concise naming conventions and encoding sufficient information about mice and image acquisition in folder and filenames.
- The available laboratory protocols were revised to ensure that their content could be understood by external collaborators, with acronyms and abbreviations explained. They were converted from .doc to Markdown (.md) files to facilitate interoperability and achieve more consistent and compact formatting.
- *Tabular metadata* within spreadsheets was missing the “codebooks” explaining the meaning of the variables. We also addressed formatting inconsistencies and machine-readability. For example, date formats, name separators, and missing value definitions were occasionally inconsistent even within the same spreadsheet. A further intervention was to combine the information from several metadata tables. We provided code and step-by-step documentation to combine information from tables with subject information (one row per mouse) and tables with multiple rows per subject, listing the available image files and their recording parameters.
- We also provided code to convert rows from metadata tables into JSON metadata files that accompany data files. One challenge was to decide which information to include in tabular and JSON metadata files. Another challenge was the level of ‘resolution’ of the JSON files. Since hundreds of image files were associated with a single mouse (i.e., one row in the metadata table), it would not have been practical to generate JSON files for each individual file. Instead, the JSON files accompanied all related files in a given folder.

## 2. Sharing metadata

The latest version of the metadata should first be shared internally, then with external collaborators, and eventually with the research community at large. Sharing these files during the “production” phase of a project, i.e., while raw data are still being generated and processed data remain subject to change, can be challenging. If the metadata files are edited by multiple collaborators, stored in multiple locations, and shared as copies, tracking the change history will be difficult and issues with outdated files are bound to arise. This can lead to uncorrected errors - or worse, unnoticed errors. It also results in inefficient communication between researchers, with file and query exchanges and duplicate efforts to keep the various metadata instances up to date. By setting up an efficient workflow for sharing metadata from the start, researchers can reduce the hurdles of making (meta)data publicly available.

### Recommendations

#### Choose a secure and version-controlled platform for sharing metadata

Especially in data-intensive projects, it is very likely that metadata will be stored and shared in a location different from the actual data files. For the storage of metadata, we recommend considering platforms with the following features: (a) centralized and synchronized online storage, (b) version control with access to history, (c) secure storage with the option to restrict online access to collaborators, (d) compliant with data governance requirements, including requirements for server location and the handling of sensitive data.

#### Consider ease of use and practicality of the platform

To enable regular use by all involved parties, the retained platform needs to be easy to use and beneficial for future projects. Hence, the platform should be (a) manageable by researchers themselves, with minimal external dependencies, (b) user-friendly for maintainers and collaborators, (c) scalable, i.e. capable of expanding to other projects, and (d) portable, i.e., material should be easily transferable to another platform if necessary - for example, an online repository enabling persistent identification of these materials (see section 3. Archiving and publishing).

Based on these features, our recommendation for researchers in the AFFORD project was to use Git repositories with version control to host their metadata and documentation, and any code involved in their production. Git repositories are widely used by programmers to develop code, but they are increasingly being proposed for scientific workflows as code becomes indispensable to research^19–21^. The popular, free and open-source Gitlab (https://about.gitlab.com) platform can be used to manage such repositories. Many academic institutions manage their own Gitlab instances, ensuring that the content is stored according to their own governance requirements. In AFFORD, we also proposed using the services of Gitlab pages to share and visualize the project metadata as webpages, allowing one to navigate the metadata, filter datasets of interest and find links to the associated data. While Git repositories are not intended for large data storage and are therefore unsuited for the storage of the actual data files, they can be integrated with a number of online data repositories.

Researchers should consider whether there are specialized data management tools better suited to their particular discipline and data type, taking into account data security requirements, existing support, cost of adoption, and community.

#### Make a decision at a group or project level

We recommend actively discussing these choices within the research group, involving all researchers participating in the project to reach some degree of consistency in the workflow implementation across the researchers. The objective is that everybody involved in the project contributes to maintaining metadata in the central shared location, and accesses that metadata as source material for using the research data. Otherwise, this effort will not succeed in preventing local duplicates, outdated copies, and scattered information.

#### Share code efficiently

We have highlighted that automating some metadata handling activities is becoming increasingly indispensable. We also acknowledged the differences in IT affinities and resources that may exist between different laboratories. An efficient way of progressively narrowing the gap between advanced programmers and non-programmers is to encourage the use of literate programming^22^, which results in documents that combine machine-readable source code with human-readable documentation. Following this paradigm, tools like Quarto^23^ and Jupyter notebooks^24^ offer the possibility to dynamically generate reports with very low additional effort beyond code writing. Dynamic reporting^25^ together with version control, is an important pillar for code reproducibility^26^. This is not only relevant for analytic code, but also for FAIR actions such as table operations as described in the subsection Learn how to perform table operations with code. Besides making these actions more reproducible, dynamic reporting facilitates the understanding and reuse of the code by collaborators who have less programming expertise.

#### Create off-boarding/on-boarding documentation

A simple and very efficient strategy to improve data curation practices in a group is to facilitate knowledge transfer between past and incoming researchers through consistent off-boarding/on-boarding documentation. These documents can include detailed information about activities related to data collection, procedures, and data management. The same FAIR principles that allow external researchers easy access to and understandability of a project’s data also apply to the on-boarding of new researchers into a group or project.

### Implementation in the reference project

In the reference project, some datasets had already been collected before more advanced data curation for open data publishing was implemented. In one use case, a researcher sought to publish a subset of datasets (linked to a report) in a specialized repository. The synchrotron images for the report had been acquired at different synchrotron facilities, each with its own metadata structure, naming conventions and folder organization. The dilemma was whether to harmonize all metadata files and folder names, creating publication-specific metadata and documentation, or to publish the data with detailed documentation explaining the inconsistencies across the different facilities. Harmonization risked reproducibility if some of the source data was used in a subsequent publication with the original metadata. Renaming files might further obscure the connection between the source dataset and the publication subset, potentially affecting associated metadata or scripts. A compromise was proposed: renaming only entries in the publication’s metadata tables that contained errors and could lead to data misinterpretation. The variables names in the tables of data sets from different facilities were also harmonized so that a single table could accommodate the information for all files in the publication. Ideally, these curation changes would be reflected in the source data. However, for that specific example, the source data amounted to about 1 petabyte, volume for which no centralized online storage was available. The source data is thus backed up on multiple physical hard drives, rendering the continuous integration of metadata changes impractical.

All groups in the reference project shared some metadata via Microsoft Sharepoint services supported by the involved universities. This is practical for all project members to some extent as it is easy to co-edit documents, access them, link them in messages, etc. However, this platform is not suited for sharing beyond the internal collaborators of the reference project. Moreover, the version history and track changes features in this platform are suited for single documents, but not for code. As a response to these needs, we developed a workflow using Gitlab repositories and Gitlab pages. The goal was to create a data catalog, i.e., a central index of the reference project, to facilitate access to all material related to the project (i.e., publications, supplementary material, metadata, and data files when hosted in online repositories). One major challenge was to make the workflow simple enough to enable all collaborators from the reference project to contribute without requiring additional programming skills or creating new dependencies. As it was not realistic for every researcher to acquire the necessary skills to curate the metadata and edit the repository, a compromise was made so that at least one or two researchers per group would take over this task for his/her group. Another challenge was to make the workflow sustainable in the long-term irrespective of the institution hosting the Gitlab instance and after the project has ended. For this, we documented the actions required to move the repository, for example, from a Gitlab instance maintained by the UZH to Gitlab.com. We also proposed a citable entry in Zenodo (https://zenodo.org/) pointing at the URL of the Gitlab page.

## 3. Archiving and publishing

When identifying the project-specific needs for data storage and archiving, it is helpful to consider general aspects such as the frequency at which the data are expected to be accessed. We can distinguish between archiving *hot* data that are used constantly, *warm* data that need regular access and *cold* data that are rarely accessed or used. Although not expected to be accessed in a long time, cold data should still be curated, unlike data considered *disposable*^27^. During the production phase of a project, researchers might generate a vast amount of data that might never be validated or become useful to the scientific questions addressed by the project. However, collecting data is expensive, usually much more than curating data, thus data disposal is usually avoided in favor of cold archiving of the entire volume of data generated. Irrespective of the data *temperature*, data archiving and/or publishing should comply with the pertinent legal data governance requirements and follow the FAIR principles as much as possible.

### Recommendations

#### Organize data folders to facilitate later archiving

The folder organization of a project in production is important for future archiving of the data, regardless whether this will be a cold archive of “everything that was acquired” or a hot archive of a subset of research data. A good organizational practice is to keep raw, unedited data clearly separated from preprocessed data, code, and analyzed data. This is relevant for the archiving process as well as for scientific reproducibility. In addition, sensitive or potentially identifying data that may not be deposited in an archive should be stored in a folder that is easy to identify and to which access restrictions can be applied if necessary.

#### Start archival curation early on

Start by producing README files describing folder contents or documenting errors or exceptions as soon as data collection begins. These files will be helpful both during data analysis and archiving. Differences in the location and infrastructure retained for cold vs. hot archiving (e.g., tapes vs. cloud storage) may lead to slight differences in folder organization and documentation needs for different archiving “temperatures”. A recommendation is to reduce these differences to a minimum, as restructuring folder organization always carries the risk of errors or data loss.

#### Use specialized utilities for moving or copying files

Over the course of a project, it is often necessary to move large volumes of data to other drives or between computers. Using utilities like rsync (https://rsync.samba.org/) for Unix-like systems or Robocopy (for Windows systems) offers several advantages over simpler file transfer methods like copy-paste or drag-and-drop. These utilities are more *efficient*, as they are designed to transfer only the differences between source and destination files (if wished), avoiding renewed transfer of the same files. They are *safer*, as they allow resumption of interrupted transfers. They can *preserve file attributes* such as timestamps, permissions, ownership and other metadata during the transfer process. They *log and handle errors*, allowing users to monitor the progress of transfers and troubleshoot any issues that may arise. We recommend that researchers consult technical experts (e.g. local IT support) before moving or copying large amounts of data to get advice on the most suitable tools for their needs.

#### Keep metadata of the storage locations

Data files and their back-up copies may be placed in different locations during the course of a project. Whether they are in server locations or on physical devices, researchers need to log where the different copies of the data can be accessed. These data location logs may be used mostly for internal purposes when a project is in production, but they still need to be curated and are important for long-term data preservation. The storage information should uniquely identify the server or the physical device containing the data. For example, the letter assigned to a network attached storage (NAS) when mounted onto a Windows computer is not sufficient information, as this is an arbitrary label that may change between devices or even between login sessions in the computer. Instead, the server address should be provided. As with any other project metadata, for this information to be useful, it needs to be maintained up to date and stored in a central location that is accessible to all researchers involved in the project.

#### Find a suitable repository

Open research data repositories aim at preserving and making data freely accessible to anyone, promoting transparency and scientific collaboration. A key requirement in a repository is that it should provide a persistent identifier to the data and metadata (see next subsection) and facilitate making the archived data “FAIR enough”. These can be determining factors when evaluating the suitability of a repository, although assessing its FAIRness may not be a straightforward task. For example, the Swiss National Science Foundation has defined a set of minimal criteria for the repositories to fulfill to be considered as FAIR enough^7^. Initiatives like the CoreTrustSeal (https://www.coretrustseal.org/) make these criteria more explicit by defining and providing certifications based on how well repositories can ensure long-term storage and accessibility of data. These certifications also try to integrate FAIR-enabling assessments, taking into account repository features that help publishing FAIRer data. For a systematic search of an adequate repository, the registry of research data repositories re3data (https://www.re3data.org/)^28^ allows to explore the available options browsing by subject, content type and country. It also allows filtering based on features like versioning, metadata standards, data licenses and many others.

#### Publish with persistent identifiers to ensure long-term findability

Research outputs need persistent identifiers (PIDs), which are globally unique, consistent and long-lasting references to objects or resources. Many online repositories and science platforms offer the possibility of obtaining a Digital Object Identifier (DOI), a commonly used type of PID, when adding new entries. A DOI can also point to a single entry with multiple version of the materials allowing to track the history of changes. However, in the *in production* phase of a project when the data and metadata are frequently being updated, making entries in DOI-enabling platforms or repositories may not be practical or feasible. If using Git repositories to share metadata as described in section 2. Sharing metadata, researchers need to think about how they may provide a persistent link to the material in the long-term. The URL of a Gitlab page, for instance, is likely to change in the future and although there are options for assigning Persistent URLs (PURL), these are not as strong as DOIs in terms of discoverability and traceability by different information systems. In AFFORD, we proposed a two-stage approach, using git to continuously catalog data and documentation (see section 2. Sharing metadata) and a well-established research repository like Zenodo (https://zenodo.org/) to describe the Git repository with the catalog and point at its URL. The DOI of the Zenodo entry provides a persistent identifier for all project outputs. If the catalog’s URL changes, researchers can easily edit the metadata of the entry in Zenodo. As some of the materials in the catalog become stable, they can be incorporated into the Zenodo record - e.g. by uploading a Gitlab release, which is a snapshot of the repository at a particular point in time. Zenodo’s DOI for citing all versions will be useful in case of errors in metadata entries, changes in data locations or other unplanned changes. This incremental archiving also makes it possible for researchers to get rid of the online catalog after project completion, the finalized catalog content remaining on Zenodo, which is ultimately the entry used to cite the project.

### Implementation in the reference project

Data archiving in this project was challenged by the fact that most of the research groups involved produced relatively large volumes of data. In addition, these data often had to be collected at different international facilities and be brought back using physical devices (hard disks). The copies of these hard disks were distributed to the three involved institutions so that collaborators could access the raw data. It was difficult to find online repositories for warm and cold archiving solutions that could handle the volume of data being generated, so in-house hard disks remained the primary means of storage. An additional challenge in this project was the non-uniform local IT resources, which hindered some of the data storage tasks (e.g., performing backups of the data on hard disks). Regarding metadata, some of the groups maintained tables with information related to the disks and data locations. The main challenge in these tables was to use controlled vocabulary (e.g., consistent variable names), to be formatted for machine readability and to have code books to ensure comprehensibility by collaborators not directly involved with those metadata.

## Discussion

In this report we shared recommendations to make research data reusable before and after publication. These recommendations are accompanied by “reality checks” in the form of insights from their implementation in our reference project. An overall impression from our support experience is that there are plenty of valid resources and approaches to improve data curation with manageable effort. However, the actual implementation of these efforts is hindered by a very fragmented landscape of tools of varying complexity, combined with large individual differences in technical skills, research cultures and perception of incentives among researchers^29^. There is also a growing awareness of the importance of roles like those of data stewards in universities, which can contribute to relieving the burden to researchers and facilitating FAIR practices in groups with less resources.

An important challenge for both researchers and data-oriented support staff is the different attitudes with respect to data management practices and their impact on the quality of research. For example, some disciplines may put more weight on publishing in prestigious journals, while others may value more the sharing of code and methodologies among peers. Concepts of reproducibility, replicability and good research practices can also be understood differently across fields and they can have multiple operational definitions^30^. Even within a research group, frictions may emerge on how to promote FAIR principles, since team members can have wide differences in digital competences and IT affinities. Again, the recommendations and insights in this report will help find a starting point even when digital competencies are sparse.

The challenges described above are also acknowledged in the final report and action plan from the European Commission expert group on FAIR data^31^. The report provides a number of specific recommendations to different stakeholders in order to change research culture and infrastructure provision to promote FAIR data. Large-scale initiatives like the European Open Science Cloud (EOSC)^32^ are vital in this transition and several communities have already made important steps in this type of national or cross-national coordinated infrastructures. Some examples are the European Plate Observing System (https://www.epos-eu.org/about-epos), the Coupled Model Intercomparison Project for climate projections (https://wcrp-cmip.org/), the Common Language Resources and Technology Infrastructure (https://www.clarin.eu/) and the Integrated Carbon Observation System (https://www.icos-cp.eu/).

Getting started with the recommendations in this report will not suffice to produce data that can be autonomously ‘understood’ and operated by machines as a digital object. According to the FAIR principles this would be the optimal but rarely achieved state^2^. The principles, nonetheless, provide a path along a continuum towards that optimal state and the recommendations in this report can help researchers set foot on that path.
